# The use of telehealth-supported stewardship activities in acute-care and long-term care settings: An implementation effectiveness trial

**DOI:** 10.1017/ice.2023.81

**Published:** 2023-12

**Authors:** Daniel J. Livorsi, Stacey Hockett Sherlock, Cassie Cunningham Goedken, Sandra Pratt, David A. Goodman, Kim C. Clarke, Hyunkeun Cho, Heather Schacht Reisinger, Eli N. Perencevich

**Affiliations:** 1 Iowa City Veterans’ Administration Health Care System, Iowa City, Iowa; 2 University of Iowa, Carver College of Medicine, Iowa City, Iowa; 3 John J. Pershing Veterans’ Administration Medical Center, Poplar Bluff, Missouri; 4 Bath Veterans’ Administration Medical Center, Bath, New York; 5 Carl Vinson Veterans’ Administration Medical Center, Dublin, Georgia; 6 University of Iowa, Department of Biostatistics, Iowa City, Iowa

## Abstract

**Background:**

We assessed the implementation of telehealth-supported stewardship activities in acute-care units and long-term care (LTC) units in Veterans’ Administration medical centers (VAMCs).

**Design:**

Before-and-after, quasi-experimental implementation effectiveness study with a baseline period (2019–2020) and an intervention period (2021).

**Setting:**

The study was conducted in 3 VAMCs without onsite infectious disease (ID) support.

**Participants:**

The study included inpatient providers at participating sites who prescribe antibiotics.

**Intervention:**

During 2021, an ID physician met virtually 3 times per week with the stewardship pharmacist at each participating VAMC to review patients on antibiotics in acute-care units and LTC units. Real-time feedback on prescribing antibiotics was given to providers. Additional implementation strategies included stakeholder engagement, education, and quality monitoring.

**Methods:**

The reach–effectiveness–adoption–implementation–maintenance (RE-AIM) framework was used for program evaluation. The primary outcome of effectiveness was antibiotic days of therapy (DOT) per 1,000 days present aggregated across all 3 sites. An interrupted time-series analysis was performed to compare this rate during the intervention and baseline periods. Electronic surveys, periodic reflections, and semistructured interviews were used to assess other RE-AIM outcomes.

**Results:**

The telehealth program reviewed 502 unique patients and made 681 recommendations to 24 providers; 77% of recommendations were accepted. After program initiation, antibiotic DOT immediately decreased in the LTC units (−30%; *P* < .01) without a significant immediate change in the acute-care units (+16%; *P* = .22); thereafter DOT remained stable in both settings. Providers generally appreciated feedback and collaborative discussions.

**Conclusions:**

The implementation of our telehealth program was associated with reductions in antibiotic use in the LTC units but not in the smaller acute-care units. Overall, providers perceived the intervention as acceptable. Wider implementation of telehealth-supported stewardship activities may achieve reductions in antibiotic use.

Infectious disease (ID) physicians are important to the implementation of antibiotic stewardship processes.^1–6^ However, ID physicians are absent from ∼25% of US hospitals, and 80% of US counties lack an ID physician.^7–9^ The absence of ID support is a barrier to the effective implementation of stewardship processes.^10,11^

Telehealth can provide ID physician expertise to resource-limited settings.^12^ Telehealth can be used to assist local stewardship strategies even when direct patient care is not provided. Studies have reported that this approach can reduce unnecessary antibiotic use and minimize antibiotic-related adverse events.^13–17^

The most effective and efficient way of deploying telehealth for stewardship activities is undefined, and the barriers to using telehealth in this manner are not well described. In this project, we assessed the implementation of telehealth-supported stewardship activities across 3 Veterans’ Affairs medical centers (VAMCs).

## Methods

### Study design

We performed a before-and-after, quasi-experimental mixed-methods study across 3 VAMCs with acute care and long-term care (LTC) units to evaluate the implementation of telehealth-supported stewardship activities. We defined a baseline (2019–2020) and an intervention period (2021). The reach–effectiveness–adoption–implementation–maintenance (RE-AIM) framework was used to guide the evaluation (Table 1).^18^

**Table 1.** RE-AIM Framework

Dimension	Level of Measurement
**Reach:** Who actually participates or is exposed to the program?	Individual
**Effectiveness:** Does the program achieve the desired benefit? Are there negative effects?	Organization
**Adoption:** Which settings are willing to initiate the program? Any why?	Organization
**Implementation:** How consistently was the program delivered? How was it adapted? How much did it cost?	Individual and Organization
**Maintenance:** To what extent does the program become a routine organizational practice?	Individual and Organization

### Site selection

Eligible VAMCs were (1) designated as rural, based on rural–urban commuting-area codes; (2) lacked onsite ID physician consultation, and (3) lacked any ID physician or ID pharmacist support for their antibiotic stewardship processes. We used data from a mandatory antibiotic stewardship survey (2016) to identify the small number of VAMCs meeting these criteria.^19^ Once an eligible site was identified, we used internal VHA resources to contact each site’s designated stewardship pharmacist. In total, 3 geographically diverse VAMCs were invited to participate in this program, and all 3 sites agreed to participate.

### Site characteristics

Intervention sites were each located in a different state. Each intervention site had both acute-care beds and LTC beds. During the 12-month intervention, average daily census ranged from 3–8 acute-care patients per VAMC, with wider variations in the average daily census on their LTC units (site 1, n = 64 patients; site 2, n = 93 patients: site 3, n = 8 patients). Two sites had an onsite microbiology laboratory, and all 3 sites lacked access to rapid molecular diagnostics. Delays in reporting microbiologic results were common. All sites had a stewardship pharmacist who had completed a 1-year clinical pharmacy residency program and a stewardship certification course but not a formal postgraduate training program in ID. These pharmacists, who had each served in their stewardship champion role for at least 4.5 years, also had several other clinical responsibilities, which varied by site. Some of these stewardship pharmacists were performing prospective audit and feedback (PAF) before the intervention, but no physician was helping them with the process. Each site had access to offsite ID consultations, which were completed by an outside ID physician through either an e-consultation or an offsite outpatient visit. Sites were able to continue to place these outside consultations throughout the project. All sites used the same electronic medical record (EMR) as the remote ID physician’s home site. Each site requested the ID physician’s credentialing information from his own VA facility. Because all sites were VHA facilities, the ID physician did not need to apply for a separate medical license from each site’s state.

### Intervention

Our intervention primarily used the following implementation strategies to improve antibiotic-prescribing.

#### Develop stakeholder relationships

During the fall of 2020, the remote ID physician met virtually (via Microsoft Teams software, Redmond, WA) with the local stewardship pharmacist, local inpatient prescribers, and hospital leadership at each of the 3 participating sites. The purpose of these meetings was to highlight the rationale for the project and to begin to build rapport.

#### Prospective audit and feedback (PAF)

During the 12-month intervention period, the remote ID physician met with the stewardship pharmacist at each site via Microsoft Teams on Monday, Wednesday, and Friday mornings to conduct telehealth-mediated PAF, or tele-PAF. Prior to each meeting, the stewardship pharmacist generated a list of all patients in acute-care and LTCs who were actively receiving antibiotics. The pharmacist was encouraged to review each patient’s medical record prior to the meeting. During the video call, the remote ID physician and the site pharmacist discussed each patient on antibiotics and looked for opportunities to prescribe antibiotics in a more evidence-based manner (eg, changing the selection, route of administration, planned duration, etc). Any recommendations were relayed in real time to the primary prescriber. Recommendations were usually communicated by telephone or in person. The remote ID physician frequently virtually joined the pharmacist in providing feedback, especially for more complicated issues or for prescribers who had shown a hesitancy to accept recommendations directly from the pharmacist. Documentation of recommendations was left to the pharmacist’s discretion. The stewardship pharmacist at sites 1 and 2 consistently documented tele-PAF recommendations in the EMR. At site 2, the remote ID physician would cosign the stewardship pharmacist’s note.

#### Education

At the end of every month, all inpatient providers at each site were e-mailed a 2-page educational sheet about a topic related to antibiotic prescribing. Topics were selected based on local needs and included the following: asymptomatic bacteriuria, *Clostridioides difficile* infections, COVID-19 management, duration of therapy for common infections, penicillin allergies, and skin and soft-tissue infections.

#### Quality monitoring

On a quarterly basis, each stewardship pharmacist was sent data on the number of tele-PAF recommendations that had been made, the type of recommendations made, and the percentage of recommendations that had been accepted. Data for these quarterly assessments were drawn from a weekly REDCap electronic survey completed by the stewardship pharmacists.

### RE-AIM outcomes

*Reach.* Reach was measured by tracking, on a weekly basis, the number of recommendations that were made and accepted using the aforementioned electronic survey as well as the names of different prescribers who received feedback.

#### Effectiveness

The primary outcome of effectiveness was inpatient days of antibiotic therapy (DOT) per 1,000 days present.^6^ Secondary outcomes were days of inpatient antibiotic-spectrum coverage (DASC) per 1,000 days present and postdischarge DOT per 100 acute-care discharges.^20,21^ DASC is a novel metric that combines both antibiotic consumption and spectrum together. Both inpatient DOT and inpatient DASC were aggregated across all 3 acute-care units and separately across all 3 LTCs monthly. Data on antibiotic use, days present, and discharges were extracted from the Corporate Data Warehouse (CDW) using the VA Informatics and Computing Infrastructure (VINCI). Inpatient antibiotic use was collected from the barcoding medication administration system, and postdischarge antibiotics were collected from the outpatient medication files. Antibiotics included all antibacterial agents administered via the following routes as defined by the National Healthcare Safety Network (NHSN): intravenous, intramuscular, digestive tract (eg, oral), or respiratory tract.^22^

Safety outcomes included acute-care, risk-adjusted, length of stay and all-cause, hospitalwide, 30-day readmission rate. These patient-centered outcomes were extracted from the Strategic Analytics for Improvement and Learning (SAIL) quarterly reports for each site.^23^

#### Adoption

Adoption was primarily measured during the preintervention virtual site visits.

#### Implementation

Implementation was measured in several ways. First, the weekly surveys were used to monitor fidelity and cost (ie, time commitment) to the project. One stewardship call per month at each site was devoted to checking on the implementation process through an iterative technique called periodic reflections.^24^ Our team’s qualitative analysts (S.H.S. and C.C.G.) attended these regularly scheduled calls to identify and discuss implementation barriers and facilitators. All calls were recorded and transcribed, and systematic notes were documented. Acceptability and adaptation as well as other implementation measures were collected through semistructured qualitative interviews with 20 key stakeholders after the 12-month tele-PAF intervention ended. Interviewees include 7 pharmacists, 5 nurse practitioners, 5 hospitalists, 2 medical directors, and the remote ID physician (Supplementary Material online).

#### Maintenance

Maintenance was primarily evaluated by sending each stewardship pharmacist or their supervisor a short e-mail with targeted questions six months after the tele-PAF intervention ended.

### Data analysis

To assess our primary and secondary effectiveness outcomes, we performed an interrupted times-series analysis using autoregressive integrated moving average (ARIMA) models.^25^ To identify the ARIMA model, a stepwise automatic forecasting algorithm was implemented in the forecast package for R version 4.2.1 statistical software.^26^

Interview data were analyzed using MAXQDA, a qualitative data program (VERBI Software, Berlin, Germany). We performed thematic content analysis via consensus-based inductive and deductive coding.^27^

The study consent process and procedures were approved by the University of Iowa Institutional Review Board (no. 202108047) and Research and Development Committee at the Iowa City VAMC.

## Results

### Reach

During the 12-month intervention period, the tele-PAF process reviewed 502 unique patients and made 681 recommendations to 24 providers across the 3 sites. These 24 providers accounted for nearly all inpatient providers prescribing antibiotics during daytime, weekday hours. Off-hour providers and nurses were generally not reached and were typically entirely unaware of the project.

**Figure 1.**
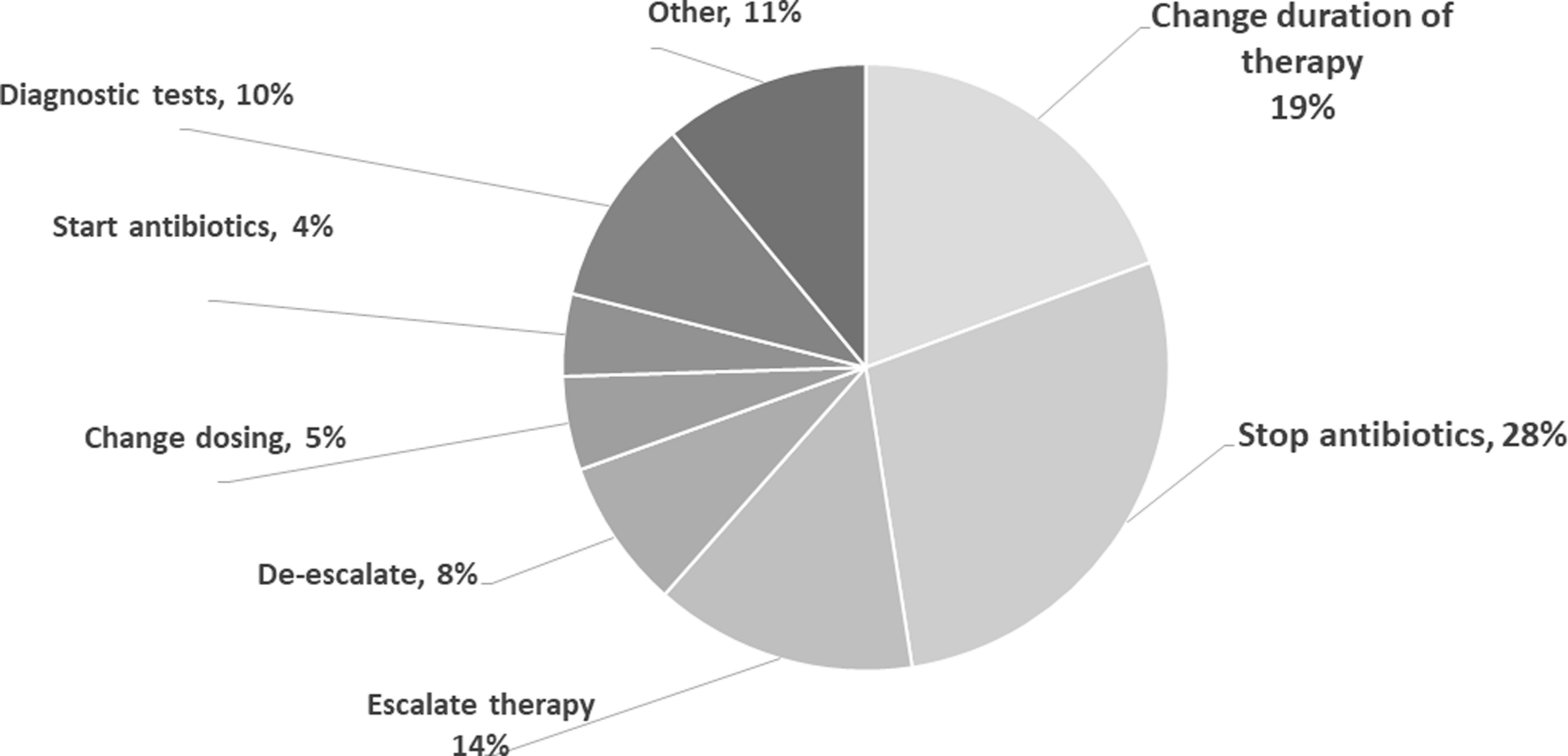
Types of recommendations made during the prospective audit-and-feedback process across the 3 participating VA medical centers, 2021 (n = 681).

Overall, 190 recommendations were made at site 1 to 8 unique providers, 380 recommendations were made at site 2 to 12 different providers, and 111 recommendations were made at site 3 to 4 providers. Seventy-seven percent of recommendations were accepted, but the overall frequency of acceptance varied across sites (site 1, 78%; site 2, 81%; and site 3, 60%). As shown in Figure 1, the most common recommendations were to stop antibiotics (28%) and change antibiotic duration (19%). Antibiotic escalation was recommended in 14% of cases.

Postintervention interviews indicated that the LTC providers were generally more receptive to feedback than acute-care providers. The LTCs had a more stable group of providers, typically nurse practitioners, who followed a cohort of patients longitudinally and, as a result, had a greater familiarity with their patients’ history. In contrast, “with the rotating schedule of the hospitalists [in acute care], it was a little bit more challenging when it was someone else who started it [the antibiotic] and recommending someone other than the lead attending to stop it” (pharmacist, site 1).

### Effectiveness

**Figure 2.**
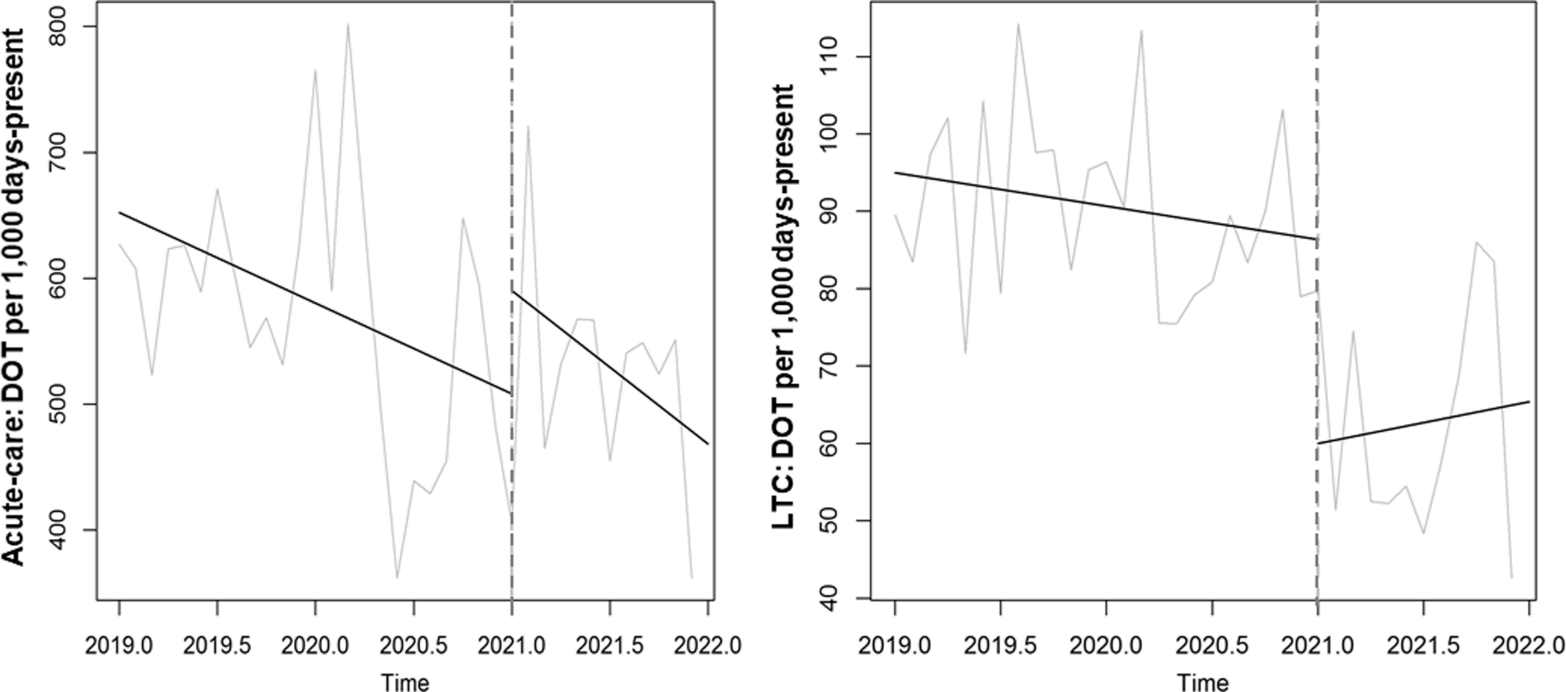
Comparison of antibiotic days of therapy (DOT) per 1,000 days-present between the baseline and intervention periods across the 3 participating VA medical centers, 2019–2021.

**Figure 3.**
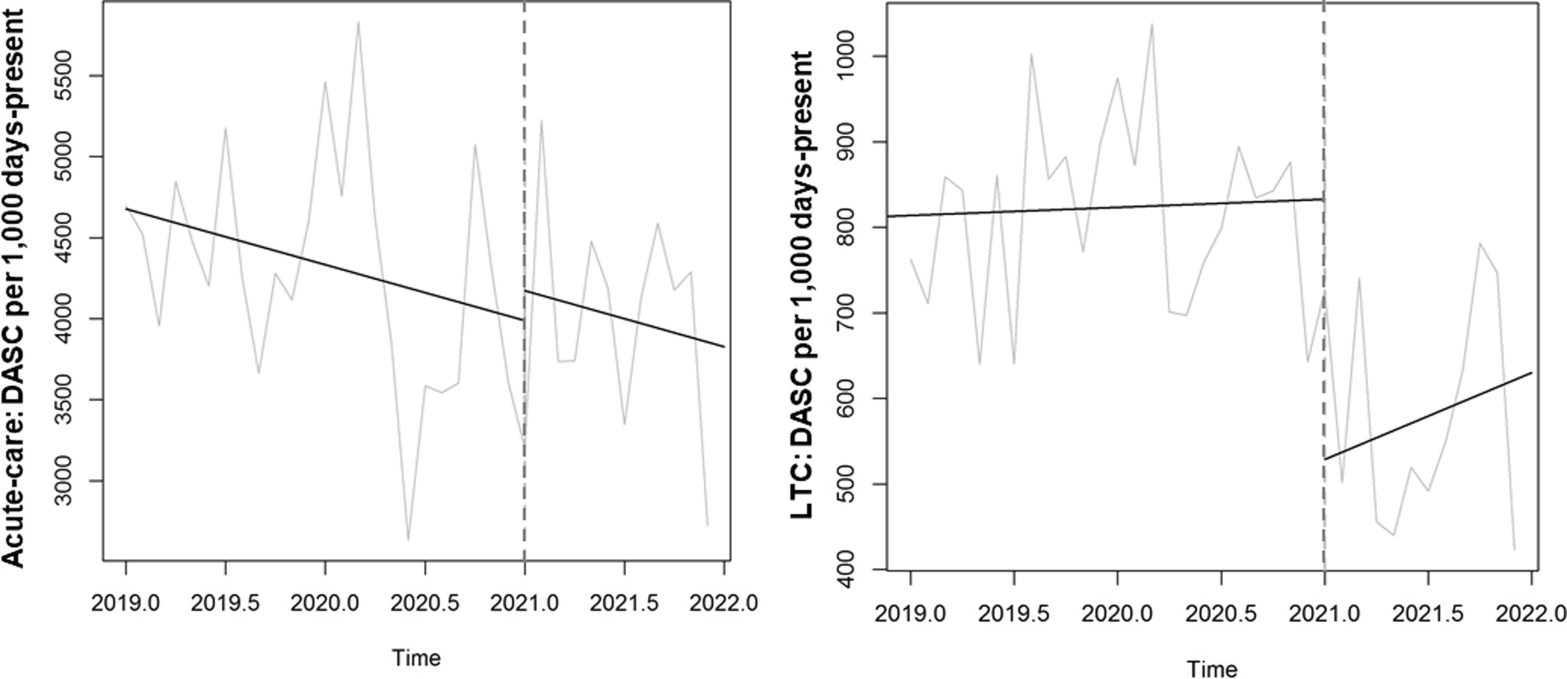
Comparison of days of antibiotic spectrum coverage (DASC) per 1,000 days present between the baseline and intervention periods across the 3 participating VA medical centers, 2019–2021.

**Figure 4.**
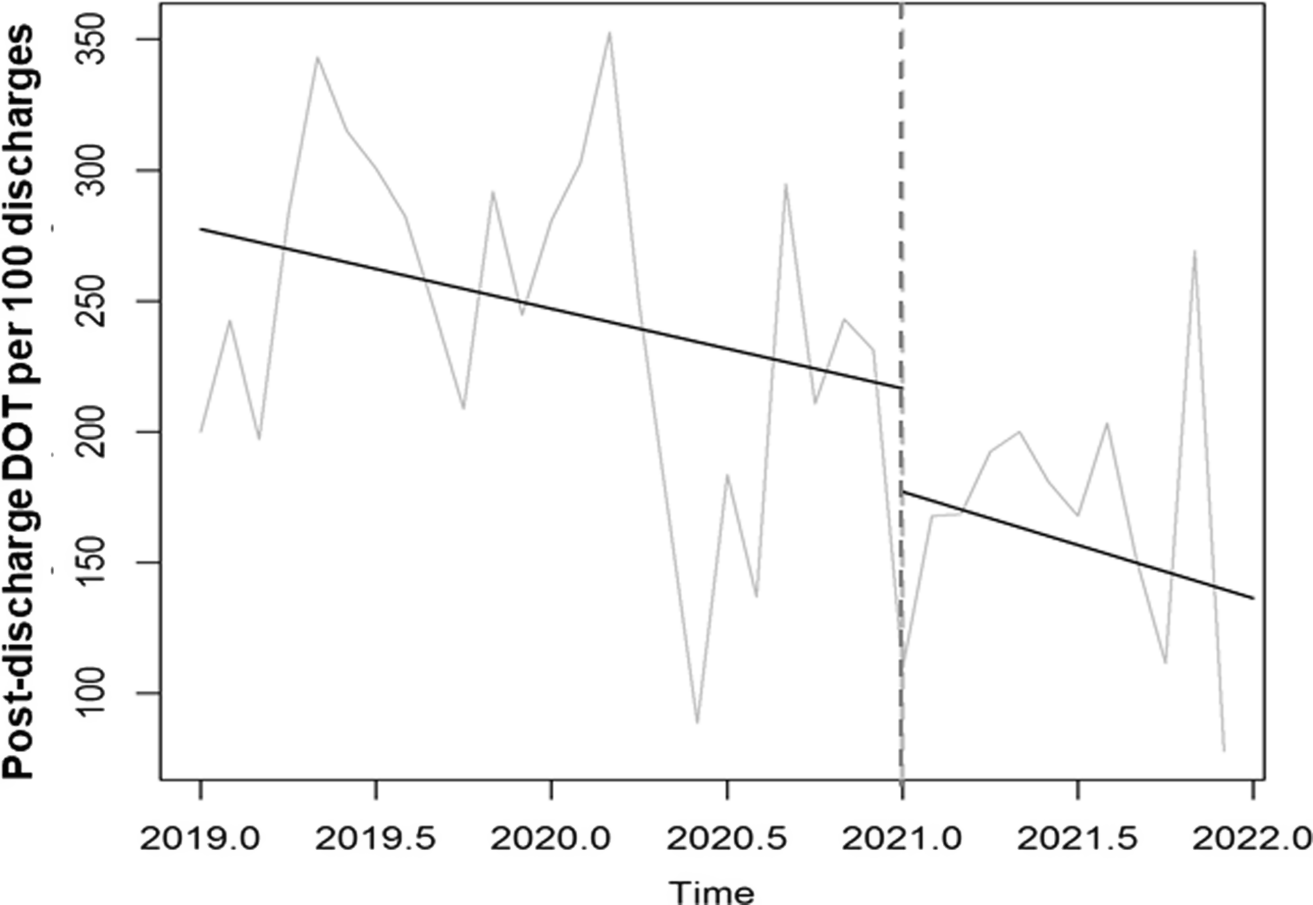
Comparison of post-discharge days of therapy (DOT) per 100 acute-care discharges between the baseline and intervention periods across the 3 participating VA medical centers, 2019–2021.

Time-series data on antibiotic use are shown in Table 2 and Figures 2, 3 and 4. After the start of the program, antibiotic DOT immediately decreased in LTC units (−26 DOT per 1,000 days present, or 30% decrease; 95% CI, 13%– 47%; *P* = .01) and then remained stable during the rest of the intervention period. There was no immediate change in antibiotic DOT in acute-care units (+82 DOT, or 16% increase; 95% CI, −10% to 42%; *P* = .22) and no change in slope during the 12-month intervention period. Different trends in antibiotic use were seen among the different sites (Supplementary Tables 1–3 and Supplementary Figs. 1–3 online).

**Table 2.** Changes in Antibiotic Use During the Baseline and Intervention Periods Across the 3 Participating Veterans’ Affairs Medical Centers

Variable	Estimate	SE	Wald	*P* Value
**Acute-care DOT per 1,000 days present**				
Slope before intervention	−6.0	2.8	4.5	.03
Immediate change due to intervention	82.3	66.6	1.5	.22
Slope change due to intervention	−4.2	8.3	0.3	.62
**LTC DOT per 1,000 days present**				
Slope before intervention	−0.4	0.3	1.4	.24
Immediate change due to intervention	−26.4	7.8	11.4	<.01
Slope change due to intervention	0.8	1.0	0.7	.41
**Acute-care DASC per 1,000 days-present**				
Slope before intervention	−28.7	19.4	2.2	.14
Immediate change due to intervention	183.9	481.6	0.2	.70
Slope change due to intervention	−0.1	58.4	<0.01	1.00
**LTC DASC per 1,000 days-present**				
Slope before intervention	1.2	2.6	0.2	.65
Immediate change due to intervention	−304.2	71.7	18.0	<.01
Slope change due to intervention	7.2	8.7	0.7	.41
**Discharge DOT per 100 discharges**				
Slope before intervention	−2.5	1.7	2.3	.13
Immediate change due to intervention	−39.6	38.9	1.0	.31
Slope change due to intervention	0.8	4.9	0.03	.86

Note. DOT, days of therapy; DASC, days of antibiotic spectrum coverage; LTC, long-term care; SE, standard error.

Antibiotic spectrum, as measured by DASC, also immediately decreased in LTC units (−304 DASC per 1,000 days present, or 37% decrease; *P* < .001) and then remained stable during the rest of the intervention period (Table 2 and Fig. 3). There were no significant changes in DASC in acute-care units. In addition, postdischarge DOT did not significantly decrease in acute-care units (Table 2 and Fig. 4).

Adjusted length of stay and adjusted 30-day readmission rates were stable or declined at each site during the intervention period (Supplementary Figs. 4 and 5 online). The volume of consultations with outside ID physicians also did not change (Supplementary Table 4 online).

### Adoption

The 3 initial sites that were eligible and invited all agreed to participate. All 3 sites requested the intervention to be implemented in both their acute-care and LTC units. The primary motivation for adoption was the lack of on-site ID support (Table 3). According to the chief of medicine at site 3, “We’re looking at this as an opportunity to get better personally, as well as benefit the veterans by preventing unnecessary use of antibiotics for prolonged durations.”

**Table 3.** Sample Quotations from Semistructured Interviews With Hospital Leaders, Pharmacists and Providers Involved in the Telehealth-Supported Antibiotic Stewardship Program Organized by the 5 Key Outcomes of the RE-AIM Framework

RE-AIM Element	Representative Quotation
Reach	“There is some consistency, kind of, with dayshift providers, but that’s not always the case [with the off-hour providers] and then too, if we have the overnight or the weekend [providers], they’re not necessarily getting face-time with the antimicrobial stewardship project or kind of knowing what’s going on because they may be here for a week and then they may not be here for another 3 months” (pharmacy leadership, site 1).
Effectiveness	“By giving them [the hospitalists] audit and feedback, then they go back and they read the literature. They look into the guidelines and then they realize [they can change their practice]” (physician administrator, site 3).
Adoption	“I think just being that we lacked an on-site ID provider, that was kind of a sell to be able to have that support for this past year” (pharmacy leadership, site 1). “I actually really loved that he [the ID physician] is doing this because being a rural facility, we sometimes don’t always have the most up-to-date providers” (physician administrator, site 2).
Implementation	Acceptability: “I think it went well. He [the ID physician] communicated mostly with our clinical pharmacist to discuss antibiotics and our infections, and then the pharmacist got the information back to me. It was useful to have. It was hard to reach an infectious disease specialist otherwise” (LTC nurse practitioner, site 1). *Facilitators to acceptability:* Collaborative communication style It “was a lot more challenging when it was me talking to them [providers]. I think the times where we still managed to be successful …was when we did have the conference call and talked about it and I think having the infectious disease expert just kind of approaching it like, you know, “Would you be willing to stop the antibiotic and monitor?” (pharmacist, site 1). “[The ID physician] would just take the time and explain what the thought process was and that seemed to make them [providers] feel better about certain recommendations” (pharmacist, site 2). “I think the way we’re doing it with [the ID physician and the pharmacist]…allows for a discussion…When I communicate my concerns [about a recommendation], I’m heard and vice versa” (hospitalist, site 2). *Barriers to acceptability: r*eluctance to accept feedback from a pharmacist: “Sometimes I think a physician might feel a little bit defensive that a pharmacist is telling [him]…what has to be done, and I’ve seen 1-2 of my providers procrastinating…It always works better to have a doctor-to-doctor conversation where things can be freely exchanged” (physician administrator, site 3). “My preference is to go with a physician [for feedback communication] because they have the clinical experience. Whereas I’ve found that … the specialty pharmacist, they will not have the clinical experience yet turn to resources” (hospitalist, site 3). *Barriers to acceptability:* lack of bedside assessments: “I think the idea of going tele from a medical perspective is not ideal. I don’t think you can get a good listening of even the lungs or see the wound, smell the wound, you know odor, drainage, things like that cannot be well visualized in a telehealth setting” (LTC nurse practitioner, site 3). “It is a limitation on the ability [of remote ID] because he’s reading my chart and he’s depending on how well did I document and my experience” (hospitalist, site 3). *However, some respondents noted that good communication between on-site providers and the remote ID physician can overcome limitations with lack of bedside assessment:* “I don’t see any of that as a barrier to using a service like this with respect to ID or any of the other sub-specialties, if I can’t communicate exactly what’s going on, then I probably shouldn’t be in this position” (hospitalist, site 1). “[The ID physician] always asks that question anyway, like ‘I’m not there, so what does the patient look like,’ you know, ‘tell me some more things that will help drive this decision’ so…I don’t feel personally I’ve had that problem [with remote ID]” (hospitalist, site 2).
Maintenance	“I would love for us to be able to continue to have that [telehealth program] not just for pharmacy, but for the entire facility as well” (Pharmacy Leadership, site 1). “I’m probably a little bit more confident just because of the experience and the frequency that we [remote ID physician and stewardship pharmacist] had contact and were able to talk about cases” (pharmacist, site 1). “I think they [the stewardship team] have taught us a lot. We are definitely overprescribing some antibiotics” (hospitalist, site 3).

### Implementation

*Fidelity*. Intervention audits were consistently performed 3 times per week at each site, except when tele-PAF team members were out of the office. However, the method for providing feedback at times varied based on the intended recipient. According to the stewardship pharmacist at site 1*, “*When I was speaking with [the ID physician] about a case, knowing who the provider was would often shape how we decided to approach it.” Because some physicians did not want to accept recommendations from the pharmacist, a 3-way telephone call was sometimes used to incorporate the remote ID physician into the conversation as well (Table 3). Certain providers were more difficult to reach, so the mode of communicating feedback had to be tailored to their work style: “It was kind of a challenge to get a hold of some of our providers. I think towards the end of the project we had a decent understanding… of more effective ways to get in touch with people” (pharmacist, site 3).

#### Acceptability

Providers generally appreciated feedback and responded favorably to collaborative discussions. According to a hospitalist at site 1, “I think it [the project] was very helpful and well received. If we could continue with that [the project], we would…. Having an ID consultant give some recommendations on the length of therapy and scaling up or down therapy is very helpful.”

The acceptability of the project was facilitated by the collaborative communication style of the ID physician (Table 3). Providers identified 2 important perceived barriers to acceptability: (1) the project’s inability to perform bedside assessments and (2) some physicians’ reluctance to act on recommendations delivered by a clinical pharmacist. Both barriers were alleviated by good communication between the providers and the ID physician (Table 3).

#### Costs

The remote ID physician devoted an average of 3.9 hours per week to tele-PAF. The mean stewardship pharmacist time commitment per week varied by site (site 1: 3.4 hours; site 2: 2.6 hours; site 3: 1.5 hours). Pharmacists at sites 1 and 2 indicated that this project was easy to incorporate into their other work responsibilities. At site 3, the pharmacist was “really involved with covering other areas due to staff leaving and us being short.” This pharmacist acknowledged that “pre-COVID, it [the project] absolutely would have been more sustainable.”

#### Maintenance

At 6 months after the project ended, none of the sites had established a contractual relationship with a remote ID physician to continue supporting stewardship activities. Sites 1 and 2 had enrolled in a VA-funded research project to further evaluate the implementation of telehealth to provide remote ID physician support. It is unclear how much their experience with tele-PAF influenced these two sites’ decision to enroll in this other project.

Although tele-PAF ended in December 2021, some benefits of the project may have been sustained (Table 3).

## Discussion

The implementation of our telehealth stewardship project was associated with reductions in antibiotic use across 3 LTC units but not in the smaller acute-care units. Overall, providers perceived the project as acceptable, and project activities were workload appropriate for stewardship pharmacists at 2 of the 3 sites.

Our telehealth project had a different effect on antibiotic use in acute-care units versus LTC units. The immediate decrease in antibiotic use in LTC units could reflect the success of engaging LTC unit stakeholders in the preimplementation phase and the ease at developing rapport with these providers once tele-PAF started. The providers in the LTC units worked every weekday and followed their patients longitudinally. In contrast, there was more frequent rotation of providers in acute-care units, which may have been a barrier to building trust. In addition, because antibiotic use was already decreasing in the acute-care units during the baseline period, there may have been fewer opportunities for improved prescribing in these settings during the intervention.

One novel aspect of our project was the use of the RE-AIM framework, which helped identify relevant outcomes to measure for program evaluation. In general, research on implementing antibiotic stewardship processes should make use of an implementation science framework.^28^ These frameworks share a common language and help more efficiently contribute to generalized knowledge about how to change behavior. The RE-AIM framework is specifically designed to evaluate health interventions, such as our telehealth project. Our use of the RE-AIM framework prompted our team to evaluate outcomes we may not have otherwise considered, such as maintenance and certain aspects of implementation. Through our implementation assessments, we gained a better understanding of how the project was adapted to meet local needs and why it was acceptable to some providers but not others. Interestingly, the barriers and facilitators to acceptability we identified have also been described in studies of stewardship performed without telehealth.^29–31^

Our project was not without limitations. First, our intervention only lasted a year, and it is unclear whether a longer intervention would have achieved larger reductions in antibiotic use. In 2 published studies, telehealth-supported antibiotic stewardship programs that lasted >2 years were associated with reductions in antibiotic use.^32,33^ The effectiveness of these programs requires developing trust and rapport between remote ID specialists and local team members, and it is possible that 1 year was insufficient for accomplishing this. Second, while all the sites were generally positive about the project, it is unclear whether the perceived value was large enough for each hospital to make a long-term funding commitment to ID telehealth. This is because sites were invited to participate without cost in a very similar telehealth project that was set to begin after the current project ended. Third, we were only able to interview a proportion of providers who received feedback as part of our tele-PAF process, and it is possible that certain perspectives about acceptability were not captured. Fourth, our analysis did not include an assessment of interfacility transfers or cost savings. Fifth, it is unclear how the COVID-19 pandemic affected our findings. Patterns of hospital staffing, antibiotic prescribing and bed use during the pandemic may have differed from the prepandemic period, which could have affected the findings of our interrupted time-series analysis. To mitigate this concern, our baseline period was 2 years in duration, roughly half of which fell during the pandemic period. Finally, our telehealth project occurred within a unique integrated healthcare system and included a remote ID physician with protected time to dedicate to this project. Although telehealth-supported stewardship can be successful under different circumstances, the implementation may be more difficult.^34^

In conclusion, using telehealth, a single, remote, ID physician was able to work effectively with 3 geographically diverse VAMCs to provide stewardship support for patients in both acute-care beds and LTC beds. Wider implementation of telehealth-supported stewardship activities may achieve further reductions in antibiotic use.

## Supplementary material

For supplementary material accompanying this paper visit https://doi.org/10.1017/ice.2023.81. click here to view supplementary material
