# Supplementary material for: The use of telehealth-supported stewardship activities in acute-care and long-term care settings: An implementation effectiveness trial
Source: Infect Control Hosp Epidemiol. 2023 Jun 14;44(12):2028–35. doi: 10.1017/ice.2023.81 (PMC10755161; doi:10.1017/ice.2023.81)
Supplement: Supplementary file 1 [file S0899823X23000818sup001.pptx]

## Slide 1
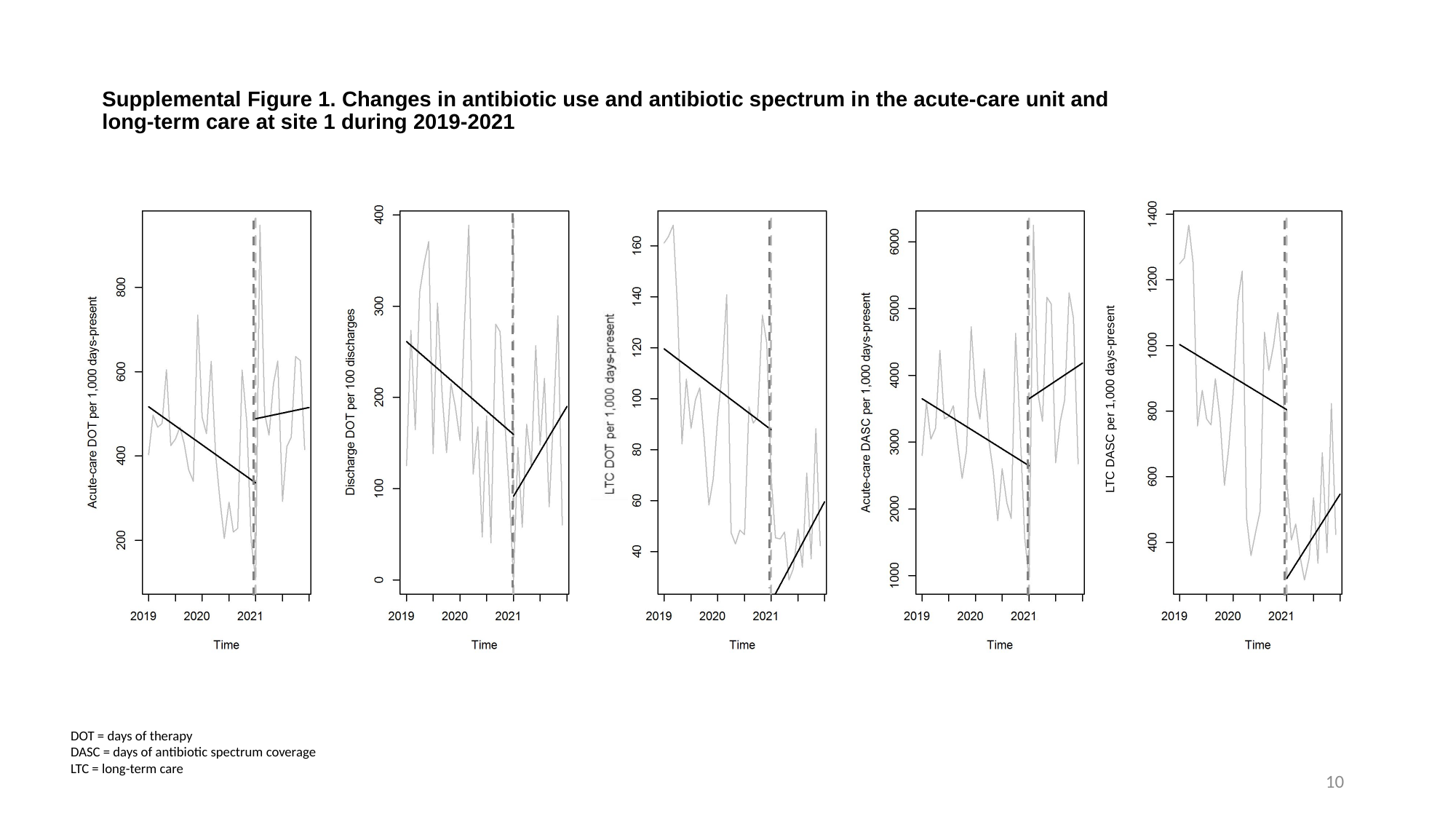

# Supplemental Figure 1. Changes in antibiotic use and antibiotic spectrum in the acute-care unit and long-term care at site 1 during 2019-2021
LTC DASC per 1,000 days-present
DOT = days of therapy
DASC = days of antibiotic spectrum coverage
LTC = long-term care
10

## Slide 2
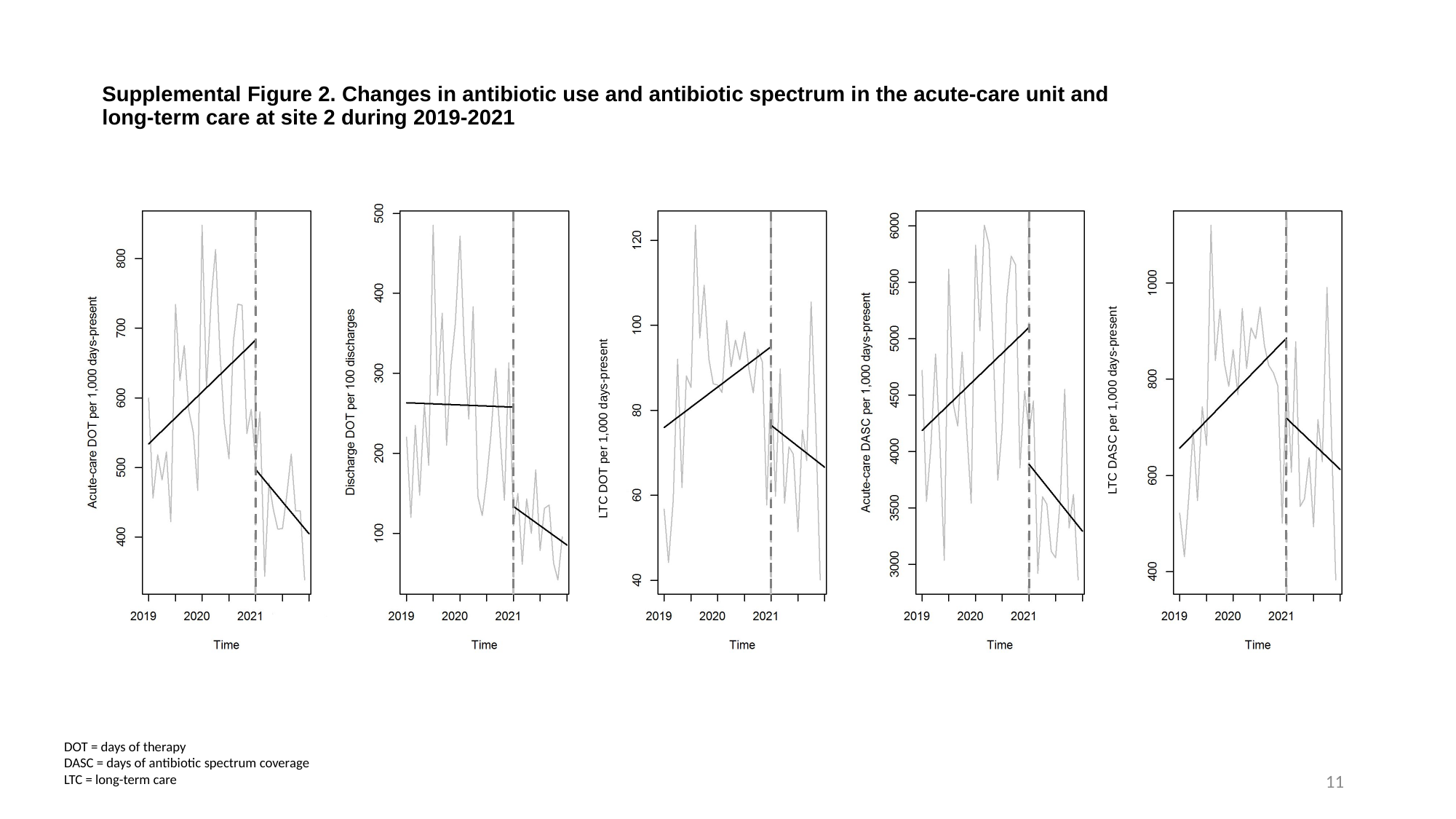

Supplemental Figure 2. Changes in antibiotic use and antibiotic spectrum in the acute-care unit and long-term care at site 2 during 2019-2021
LTC DASC per 1,000 days-present
LTC DOT per 1,000 days-present
DOT = days of therapy
DASC = days of antibiotic spectrum coverage
LTC = long-term care
11

## Slide 3
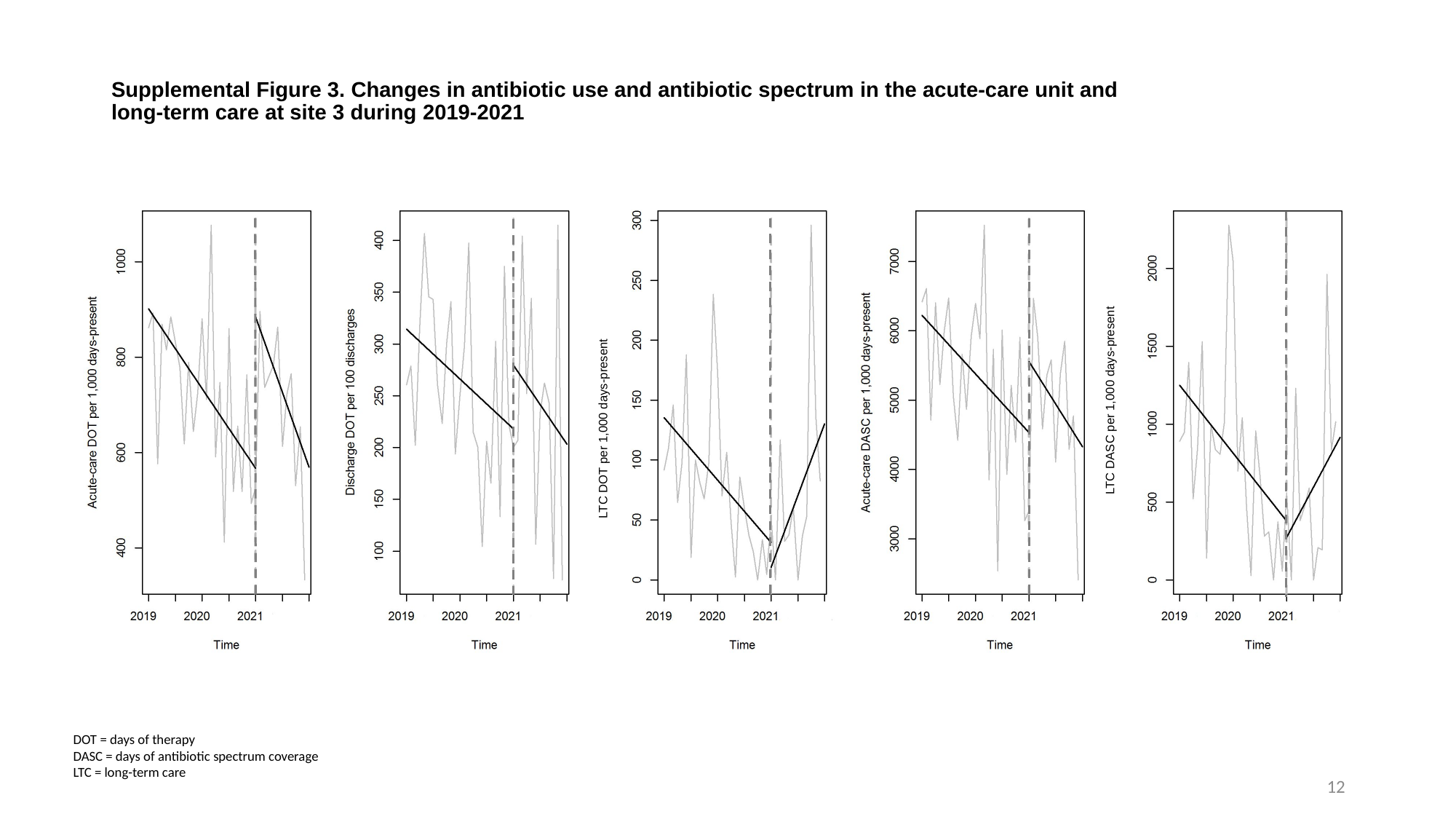

Supplemental Figure 3. Changes in antibiotic use and antibiotic spectrum in the acute-care unit and long-term care at site 3 during 2019-2021
LTC DASC per 1,000 days-present
LTC DOT per 1,000 days-present
DOT = days of therapy
DASC = days of antibiotic spectrum coverage
LTC = long-term care
12

## Slide 4
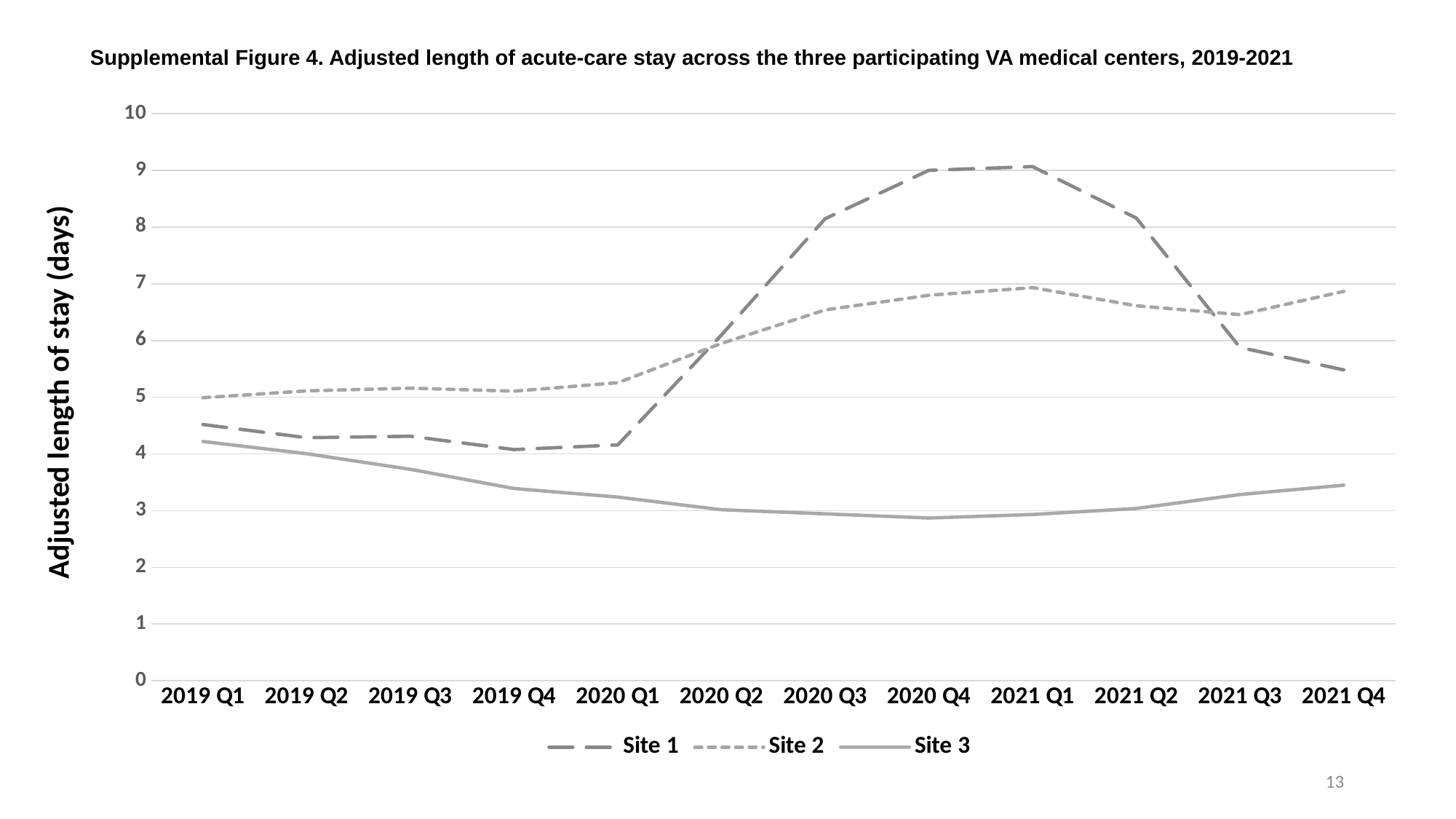

Supplemental Figure 4. Adjusted length of acute-care stay across the three participating VA medical centers, 2019-2021
### Chart
| Category | | | |
|---|---|---|---|
| 2019 Q1 | 4.519 | 4.992 | 4.219 |
| 2019 Q2 | 4.286 | 5.112 | 4.004 |
| 2019 Q3 | 4.312 | 5.161 | 3.729 |
| 2019 Q4 | 4.077 | 5.107 | 3.39 |
| 2020 Q1 | 4.159 | 5.256 | 3.238 |
| 2020 Q2 | 6.098 | 5.95 | 3.016 |
| 2020 Q3 | 8.148 | 6.54 | 2.943 |
| 2020 Q4 | 9.001 | 6.798 | 2.87 |
| 2021 Q1 | 9.067 | 6.933 | 2.932 |
| 2021 Q2 | 8.16 | 6.613 | 3.038 |
| 2021 Q3 | 5.878 | 6.456 | 3.284 |
| 2021 Q4 | 5.482 | 6.867 | 3.449 |Adjusted length of stay (days)
13

## Slide 5
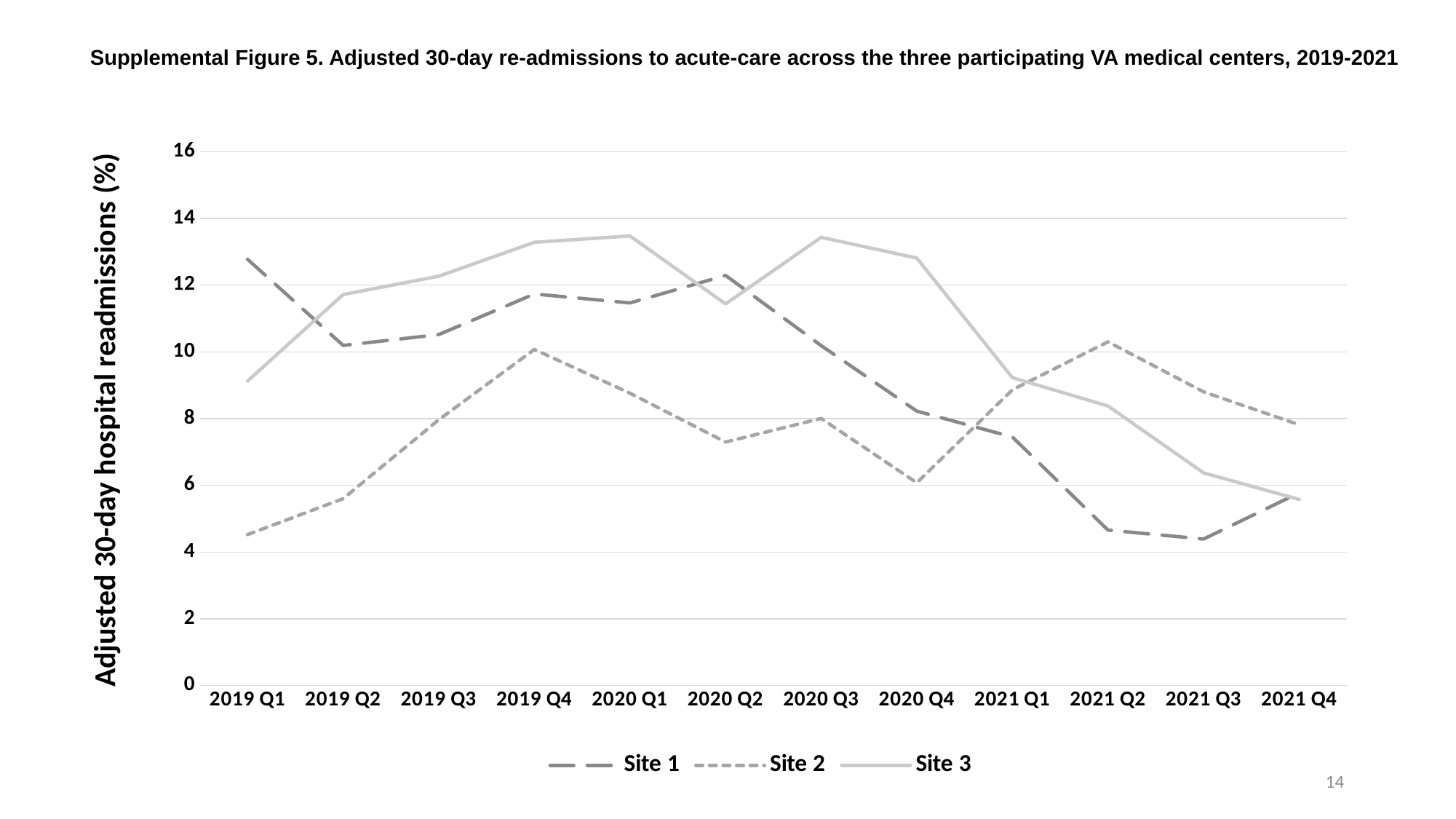

Supplemental Figure 5. Adjusted 30-day re-admissions to acute-care across the three participating VA medical centers, 2019-2021
### Chart
| Category | | | |
|---|---|---|---|
| 2019 Q1 | 12.778 | 4.522 | 9.126 |
| 2019 Q2 | 10.193 | 5.599 | 11.719 |
| 2019 Q3 | 10.519 | 7.967 | 12.268 |
| 2019 Q4 | 11.736 | 10.073 | 13.285 |
| 2020 Q1 | 11.468 | 8.761 | 13.475 |
| 2020 Q2 | 12.293 | 7.297 | 11.439 |
| 2020 Q3 | 10.188 | 8.005 | 13.435 |
| 2020 Q4 | 8.225 | 6.074 | 12.814 |
| 2021 Q1 | 7.448 | 8.863 | 9.227 |
| 2021 Q2 | 4.657 | 10.302 | 8.379 |
| 2021 Q3 | 4.389 | 8.803 | 6.371 |
| 2021 Q4 | 5.776 | 7.814 | 5.576 |Adjusted 30-day hospital readmissions (%)
14
